# Exercise‐dependent increases in protein synthesis are accompanied by chromatin modifications and increased MRTF‐SRF signalling

**DOI:** 10.1111/apha.13496

**Published:** 2020-05-30

**Authors:** Francesca Solagna, Leonardo Nogara, Kenneth A. Dyar, Franziska Greulich, Ashfaq A. Mir, Clara Türk, Theresa Bock, Alessia Geremia, Martina Baraldo, Roberta Sartori, Jean Farup, Henriette Uhlenhaut, Kristian Vissing, Marcus Krüger, Bert Blaauw

**Affiliations:** ^1^ Venetian Institute of Molecular Medicine (VIMM) Padova Italy; ^2^ Department of Biomedical Sciences University of Padova Padova Italy; ^3^ Molecular Endocrinology, Institute for Diabetes and Cancer (IDC) Helmholz Zentrum Munich Helmholtz Diabetes Center (HMGU) Munich Germany; ^4^ Research laboratory for Biochemical Pathology Department of Clinical Medicine & Department of Biomedicine Aarhus University Aarhus Denmark; ^5^ Chair for Metabolic Programming TUM School of Life Sciences ZIEL‐Institute for Food & Health Freising Germany; ^6^ Department of Public Health, Section for Sport Science Aarhus University Aarhus Denmark; ^7^ Institute for Genetics Cologne Excellence Cluster on Cellular Stress Responses in Aging‐Associated Diseases (CECAD) University of Cologne Cologne Germany

**Keywords:** exercise, histone phosphorylation, protein synthesis, serum response factor, skeletal muscle

## Abstract

**Aim:**

Resistance exercise increases muscle mass over time. However, the early signalling events leading to muscle growth are not yet well‐defined. Here, we aim to identify new signalling pathways important for muscle remodelling after exercise.

**Methods:**

We performed a phosphoproteomics screen after a single bout of exercise in mice. As an exercise model we used unilateral electrical stimulation in vivo and treadmill running. We analysed muscle biopsies from human subjects to verify if our findings in murine muscle also translate to exercise in humans.

**Results:**

We identified a new phosphorylation site on Myocardin‐Related Transcription Factor B (MRTF‐B), a co‐activator of serum response factor (SRF). Phosphorylation of MRTF‐B is required for its nuclear translocation after exercise and is accompanied by the transcription of the SRF target gene *Fos*. In addition, high‐intensity exercise also remodels chromatin at specific SRF target gene loci through the phosphorylation of histone 3 on serine 10 in myonuclei of both mice and humans. Ablation of the MAP kinase member MSK1/2 is sufficient to prevent this histone phosphorylation, reduce induction of SRF‐target genes, and prevent increases in protein synthesis after exercise.

**Conclusion:**

Our results identify a new exercise signalling fingerprint in vivo, instrumental for exercise‐induced protein synthesis and potentially muscle growth.

## 1 INTRODUCTION

Skeletal muscle is a highly plastic tissue that undergoes major alterations in form and function in response to variations in activity, load, nutrition and hormone status.^1^ Depending on intensity, duration and frequency, exercise can increase either resistance to fatigue or muscle strength. Resistance exercise is characterized by a low frequency of exercise repetitions with high mechanical stress (load), consequently leading to increased muscle mass and power output. However, which are the early signalling events required to increase protein synthesis rates, eventually leading to muscle growth, is not clear.

In order to better understand the transcriptional profile underlying this growth response, we recently analysed the gene expression changes accompanying muscle hypertrophy in mice.^2^ We observed that Serum Response Factor (SRF) and Activator Protein 1 (AP1, a FOS‐JUN heterodimer) were among the main transcription factors activated after an acute hypertrophic stimulus. Interestingly, also in humans, resistance exercise is accompanied by alterations in expression levels of SRF and its transcriptional coactivators, the Myocardin‐Related Transcription Factors (MRTFs) A and B.^3^ The ability of MRTFs to modulate SRF activity is linked to their nuclear localization, which in vitro is highly linked to the polymerization status of actin.^4^ Indeed, due to the presence of their RPEL actin binding domains, MRTFs bind monomeric G‐actin and are normally retained in the cytoplasm. However, upon mechanical stress dependent actin polymerization into filamentous F‐actin, MRTFs re‐localize to the nucleus increasing SRF transcriptional activity.^5^ The importance of this pathway for skeletal muscle is clear from transgenic models, as mice lacking both MRFT‐A and MRTF‐B from skeletal muscle show perinatal lethality.^6^ In addition, inducible skeletal‐muscle targeted deletion of SRF in adult mice prevents overload induced hypertrophy,^7^ suggesting a role of SRF also for growth of adult muscle.

Full activation of SRF‐dependent transcription in vitro requires the combination of MRTFs translocation to the nucleus and chromatin unfolding mediated by a sequential cascade of activating histone marks, starting with the phosphorylation of histone 3 on serine 10 (H3S10ph) around the transcription start site of target genes.^8^ The critical importance of this histone mark is intriguing, as in most cell types H3S10ph is mediated by the Mitogen‐Activated Protein (MAP) kinase cascade, an important signalling pathway activated after eccentric contractions.^9^ However, it is not known if and how this regulation occurs also in vivo, and which stimuli could activate SRF signalling in skeletal muscle.

Here, in order to determine how resistance exercise affects muscle growth and potentially SRF signalling, we performed an unbiased, quantitative phosphoproteomics analysis in mice subjected to a bout of 50 high intensity eccentric contractions and identified a previously unknown exercise‐dependent phosphorylation site on MRTF‐B, required for its nuclear localization in vivo. Moreover, we show that MAP kinase‐dependent H3S10ph occurs on SRF target genes upon the same bout of exercise and is required for the increase in protein synthesis normally observed after eccentric contractions. Noteworthy, human muscle biopsies after high intensity exercise show MAPK activation in human muscle in concomitance with H3S10ph. In conclusion we suggest the existence of a conserved chromatin remodelling pathway that, together with MRTF‐dependent SRF activation, is necessary for protein synthesis after exercise.

## 2 RESULTS

### 2.1 A single series of high intensity contractions leads to major changes in the muscle phosphoproteome

**Figure 1:**
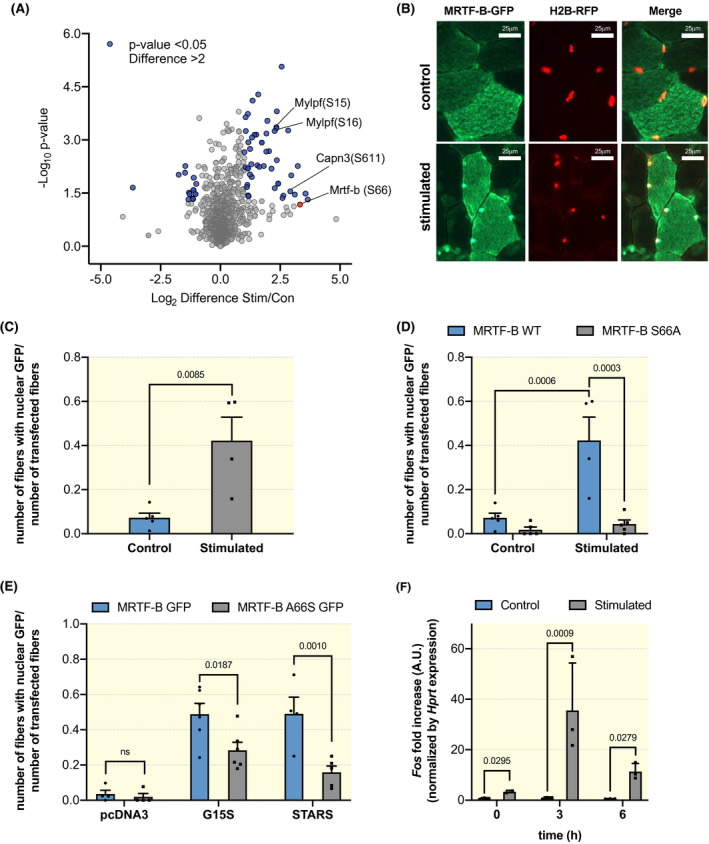
Phosphorylation of MRTF‐B on serine 66 is required for its nuclear accumulation after stimulation. A, Volcano plot showing the alterations in the phosphor‐proteome after a single bout of 50 eccentric contractions. In blue are highlighted the phosphorylation sites, which show a significant difference as compared to the contralateral control muscle (*P* < .05, paired *t*‐test), and at least a twofold change in intensity. B, Representative images of fibres co‐transfected with MRTF‐B‐GFP and histone‐red (images taken at 63x). As can be seen in the upper panel, in control muscles MRTF‐B‐GFP is predominantly localized in the cytoplasm of transfected fibres. In the lower panels a nuclear accumulation of MTRF‐B‐GFP after 50 eccentric contractions is shown through colocalization with a plasmid coding for histone‐RFP. C, Quantification of the number of transfected fibres showing nuclear localization normalized to the total number of transfected fibres in control and stimulated muscles. Muscles were stimulated 10 days after electroporation (n = 4 muscles, at least 10 transfected fibres per muscle; unpaired Student *t*‐test, *P* = .0085). D, Quantification of the nuclear translocation of MRTF‐B‐GFP after stimulation for both the wild type constructs as well as the mutant construct in which serine 66 is substituted by an alanine. No increase in nuclear localization was observed with the mutant construct after stimulation (n = 4 muscles, at least 10 transfected fibres per muscle; ordinary two‐way ANOVA, interaction *P* = .0034; Tukey's multiple comparison test). E, Co‐transfection of MRTF‐B‐GFP WT with the activating actin mutant G15S or with Striated muscle activator of Rho signalling (STARS) leads to a nuclear accumulation in 50% of transfected fibres. Co‐transfection of G15S and STARS with the mutant MRTF‐B‐S66A‐GFP significantly reduces nuclear localization (n = 4 muscles, at least 10 transfected fibres per muscle; ordinary two‐way ANOVA, interaction *P* = .0374, Sidak's multiple comparison test). F, Expression level of the SRF target gene *Fos* at various timepoints after eccentric contractions (n = 3 muscles per timepoint, RM two‐way ANOVA, interaction *P* = .0483, paired *t*‐test)

High intensity resistance exercise regimens can stimulate a muscle remodelling programme, which over time induces an increase in muscle mass.^10^, ^11^, ^12^ Here, we analyse the signalling changes that occur in vivo after 50 eccentric contractions and determine how they affect muscle remodelling. Muscle histology at different time points after the stimulation protocol do not show signs of necrotic fibres or infiltration (Figure S1A). Importantly, while muscle force declined during the stimulation bout, this decline was no longer observed at different time points after stimulation (Figure S1B). These results strongly suggest that the mild eccentric component of our stimulation protocol is not inducing muscle damage. To identify signalling changes associated with high intensity eccentric exercise, we performed a quantitative phosphoproteomics analyses immediately after a single bout of 50 unilateral eccentric contractions in gastrocnemius muscle. The stimulated and the contralateral non‐stimulated muscles were enriched for phosphorylated peptides and analysed by mass spectrometry (workflow in Figure S1C). In order to have a quantitative measurement of the changes in phosphopeptides, muscle lysates were mixed with those obtained from the muscles of SILAC mice.^13^ In Figure 1A we show a volcano plot of the fold changes in phosphorylation after eccentric contractions. We identified over 5000 different phosphopeptides, of which 1898 were quantified as they were found at least twice in each condition (Table S2) The relative distribution of serine/threonine/tyrosine phosphorylations is in line with the changes observed in most cell types (Figure S1D).

### 2.2 Phosphorylation of MRTF‐B on serine 66 is required for its nuclear accumulation after eccentric contractions

One of the proteins with the biggest fold change in phosphorylation after high intensity exercise is the transcriptional co‐activator MRTF‐B. After eccentric contractions we observed a 3,5 log2‐fold increase in phosphorylation on serine 66 (Figure 1A; Figure S1E). Since this phosphorylation site is right next to one of the RPEL domains of MRTF‐B, important for its nucleo‐cytoplasmic shuttling, we wondered if MRTF‐B phosphorylation could affect its localization. First, we checked localization of MRTF‐B in basal condition and after 50 eccentric contractions. We electroporated MRTF‐B‐GFP in tibialis anterior muscles and we found that MRTF‐B‐GFP is predominantly localized in the cytoplasm under basal conditions. Electrical stimulation leading to eccentric contractions induced its nuclear translocation in 43 ± 13% of transfected fibres (Figure 1B,C). To understand if this increase in nuclear MRTF‐B could be linked to its phosphorylation on serine 66, we generated a phospho‐null mutant, in which we substituted serine 66 with an alanine residue (MRTF‐B‐S66A‐GFP). Interestingly, MRTF‐B‐S66A‐GFP showed cytoplasmic localization both under basal conditions and after eccentric contractions, suggesting that phosphorylation of Serine 66 is required for activity‐induced nuclear accumulation of MRTF‐B.

We next explored the mechanism potentially linking serine 66 phosphorylation in MRTF‐B and actin dynamics. We used two different well‐established methods to induce MRTF nuclear accumulation. In the first we electroporated MRTF‐B together with a plasmid coding for Striated muscle Activator of Rho Signalling (STARS), which is a muscle‐specific actin‐binding protein known to reduce G‐actin levels, thereby also reducing actin‐MRTF binding. Co‐transfection of MRTF‐B and STARS lead to a significant increase in nuclear MRTF‐B (Figure 1E). Interestingly, co‐transfection of MRTF‐B‐S66A‐GFP with STARS showed an impairment in MRTF‐B‐S66A‐GFP nuclear accumulation, suggesting that phosphorylation of serine 66 is required for a full translocation of MRTF‐B to the nucleus. Next, we examined the effect of an activating actin mutant, G15S, which does not require changes in actin polymerization for the nuclear accumulation of MRTFs. Co‐transfection of G15S with MRTF‐B lead to nuclear localization of MRTF‐B in 49 ± 6% of transfected fibres. Interestingly, co‐transfection of MRTF‐B‐S66A with G15S leads to a significant reduction in the percentage of transfected fibres expressing nuclear MRTF‐B.

To understand if this nuclear accumulation of MRTF‐B is also accompanied by an increased transcription of an SRF target gene, we evaluated the expression levels of *Fos* at different time points after eccentric contractions. As can be seen in Figure 1F, we observed a very rapid and transient increase in transcription of the SRF target gene, *Fos*, suggesting an increased SRF transcriptional activity in concomitance with MRTF translocation.

### 2.3 Eccentric contractions induce histone phosphorylation on SRF target genes

**Figure 2:**
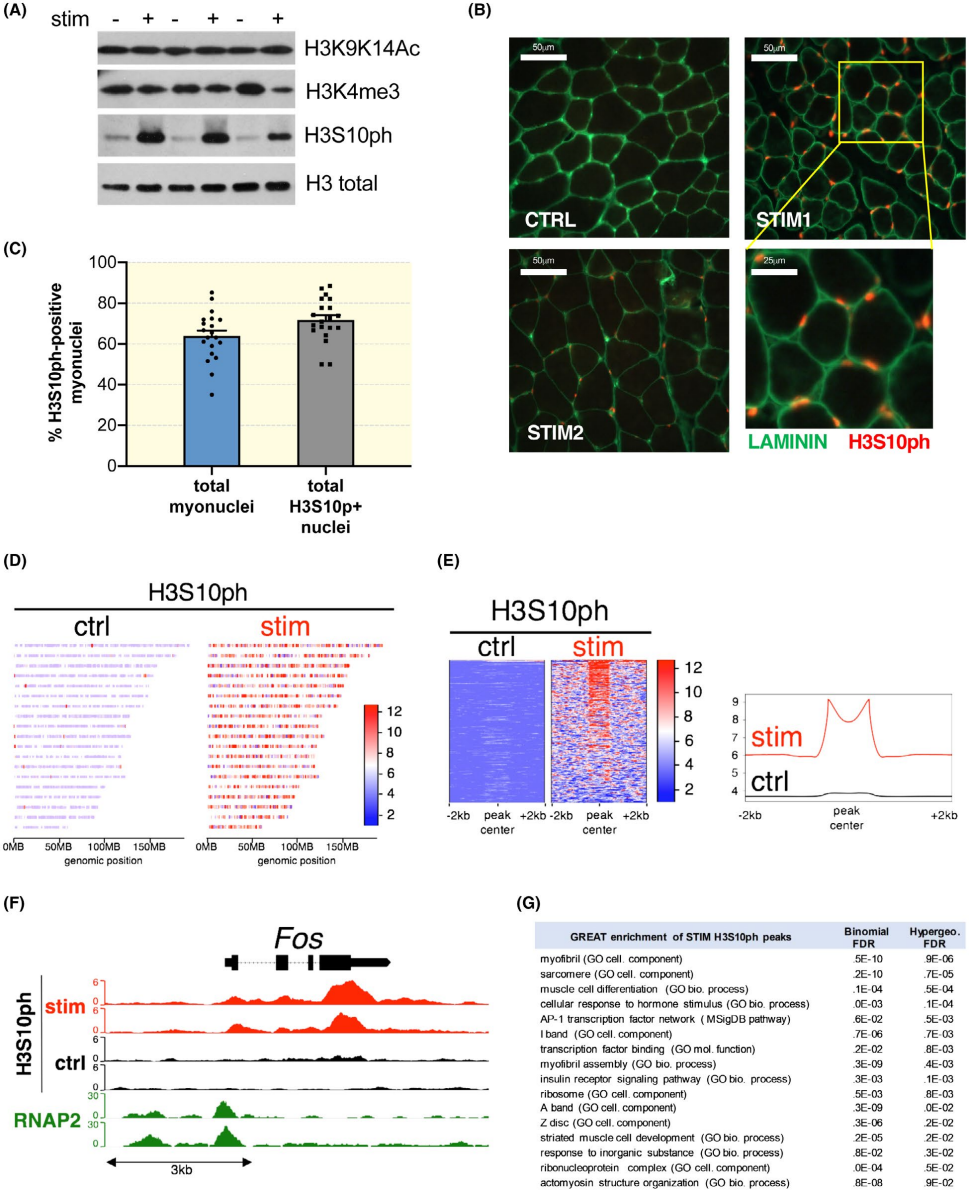
High intensity exercise leads to rapid histone phosphorylation on SRF target genes. A, Three different histone modifications linked to activation of gene expression were examined by western blotting, before and after 50 eccentric contractions. Only H3S10ph showed a marked change in stimulated muscles. B, Immunohistochemistry images showing that many of the H3S10ph‐positive nuclei are inside the basal muscle lamina (green), suggesting they are either myonuclei or satellite cells (images taken at 40x). C, Quantification of the percentage of H3S10ph‐positive myonuclei on the total amount of myonuclei (left bar). The right bar indicates the percentage of myonuclei within all H3S10ph‐positive nuclei. Total amount of nuclei was determined using a DAPI staining (n = 4 muscles, whole muscle sections analysed, unpaired Student *t*‐test, *P* = .0329). D, Heatmap showing H3S10ph enrichment across all murine autosomes (10kb bins) in contralateral unstimulated control and stimulated muscles. E, Heatmap & histogram of H3S10ph genomic binding sites (peaks). Scale indicates normalized unique tag counts. Contralateral unstimulated muscle used as control. F, Genome browser view of *Fos* gene showing H3S10ph enrichment across the gene body. RNA polymerase II (RNAP2) marks the promoter. Contralateral unstimulated muscle used as control. G, Functional enrichment analysis (GREAT) of 400 stim‐specific reproducible peaks

It was recently shown that increased SRF activity in vitro is linked to a histone modification cascade starting with the phosphorylation of serine 10 on histone 3 (H3S10ph).^8^ To understand if a similar histone remodelling occurs in skeletal muscle after acute exercise, we analysed different histone modifications, that is, H3‐K9K14ac, H3K4me3 and H3S10ph, comparing control and stimulated muscles. Immediately after stimulation, no significant changes were found in H3‐K9K14ac and H3K4me3 (Figure 2A). However, we observed that electrical stimulation induced a marked increase in H3S10ph, when compared to the low basal level in control muscles.

Since this histone mark is linked to cell proliferation, we performed an immunohistochemistry analysis to determine if H3S10ph occurs inside myonuclei, not in interstitial cells. As can be seen in Figure 2B, most positive nuclei (in red) are localized inside the muscle lamina and are, therefore, either myonuclei or satellite cells. Quantification of the positive nuclei showed that 64 ± 4% of all myonuclei are positive for H3S10ph, while 74 ± 4% of all H3S10ph‐positive nuclei are myonuclei. To rule out the possibility that this increased H3S10ph was an artefact of electrical stimulation we also performed eccentric contractions in a more physiological context by performing downhill treadmill running. Downhill running is also able to increase H3S10ph, even though the intensity is less than that observed after electrical stimulation, in which all motor units are recruited simultaneously (Figure S2A). H3S10ph is considered a priming step for subsequent modifications on nearby amino acids.^14^ In particular, the acetylation of lysine 14 on the same histone tail allows the recruitment of 14‐3‐3 proteins and activation of gene transcription. Interestingly, immediately after one bout of 50 eccentric contractions, we found a strong increase not only in H3S10ph, but also in H3S10phK14ac (Figure S2B), which slowly decreased over time (Figure S2C).

To identify H3S10 phosphorylation marks on a genome‐wide scale, we performed in vivo chromatin immunoprecipitation followed by next generation sequencing (ChIP‐seq). We mapped genomic H3S10ph marks in stimulated muscles immediately after 50 eccentric contractions, and compared them with contralateral, unstimulated muscles from the same mice. In agreement with H3S10ph western blots and immunostaining, we noticed a relatively low basal H3S10ph signal in the contralateral unstimulated muscles, yet a widespread enrichment of H3S10ph marks after eccentric contractions (Figure 2D), reflecting global accumulation across the genome. We also noticed H3S10ph broadly marked target domains with a bimodal distribution in the stimulated muscles (Figure 2E). Inspection of H3S10ph marks with the highest signal revealed specific marks across gene bodies of immediate early genes, especially those coding for the activator protein‐1 (AP‐1) heterodimers like *Fos* (Figure 2F), *Jun* (Figure S2E), and *Egr1* (Figure S2F), which we further confirmed by functional enrichment analysis (Figure 2G).

### 2.4 Histone phosphorylation and increases in protein synthesis after stimulation requires MSK1

**Figure 3:**
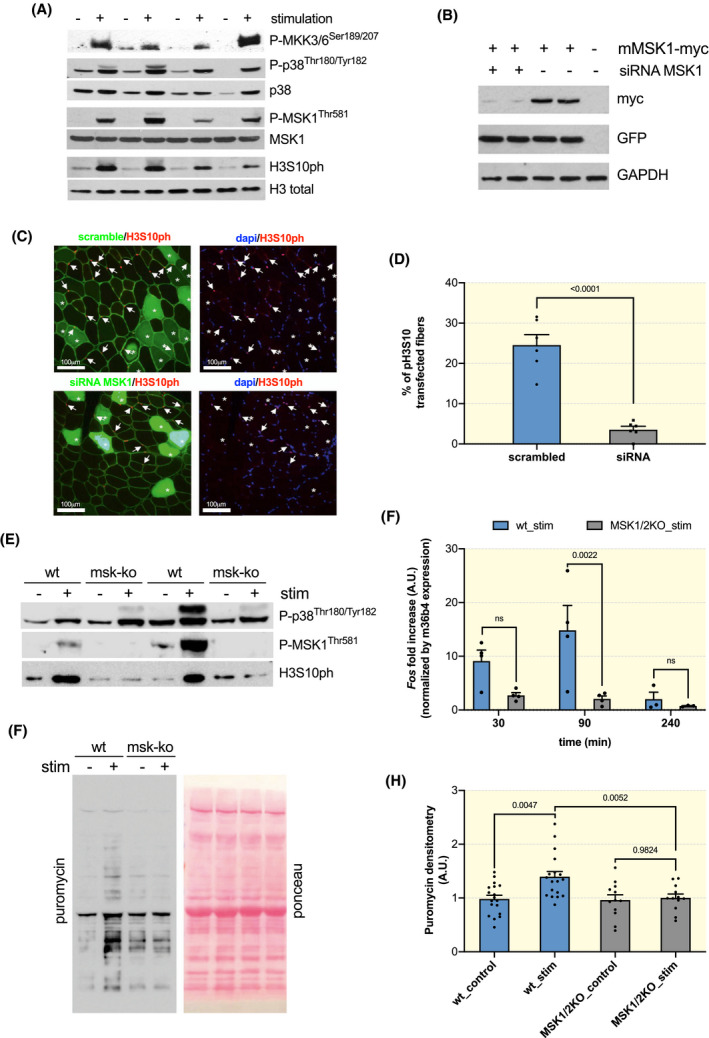
MSK1‐dependent H3S10 phosphorylation accompanies induction of SRF target genes and protein synthesis increases after exercise. A, Phosphorylation and activation of the MAPK pathway members MKK3/6, p38, and MSK1 immediately after 50 eccentric contractions, but not in contralateral non‐stimulated muscles. B, Efficiency of the siRNA for MSK1 in Hela cells transfected with a mouse MSK1‐myc and PE‐GFP. C, Electroporation of siRNA‐scrambled‐GFP and siRNA‐MSK1‐GFP in TA muscles 10 days before performing 50 eccentric contractions. In the upper images fibres transfected with the scrambled sequence are shown. Both transfected and non‐transfected fibres show H3S10ph‐positive nuclei. In the lower images fibres were transfected with siRNA for MSK1 and did not show nuclear H3S10ph after stimulation. Importantly the non‐transfected fibres in the same muscle do show H3S10ph (images taken at 20x). D, Quantification of the number of transfected fibres with H3S10ph after eccentric contractions show a very significant reduction in H3S10ph‐positive myonuclei after transfection with siRNA for MSK1 (unpaired Student *t*‐test, *P* < .0001) E, Western blot showing no H3S10ph, but increased P‐p38, in MSK1/2 k.o. mice after stimulation. Sample load 70 μg. F, Reduced transcription of *Fos* after stimulation in MSK1/2 k.o. mice (n = 4; ordinary two‐way ANOVA, interaction *P* = .0391, Tukey's multiple comparison test, 30 minutes n comparison *P* = .7622, 90min comparison *P* = .0022, 240 minutes comparison *P* = .8198). G, Representative blot for puromycin incorporation in the stimulated and contralateral non‐stimulated muscle in both wild‐type and MSK1/2 k.o. mice. H, Quantification of protein synthesis rates in stimulated and contralateral unstimulated control muscles from wt and MSK1/2 k.o. mice (n = 18 wt; n = 12 ko; RM two‐way ANOVA, interaction genotype/stimulation *P* = .0473; Sidak's multiple comparison, paired *t*‐test)

Considering that H3S10ph is such an early event and has been linked to the induction of SRF target genes in vitro, we aimed at identifying the kinase responsible for this phosphorylation. Since the MAP Kinase (MAPK) pathway is rapidly activated after exercise,^15^ we first examined its activation levels after our stimulation protocol. We found a strong increase in the phosphorylation and therefore activation of the dual specificity kinases MKK3/6, p38, and MSK1 that closely matched the phosphorylation of H3S10 (Figure 3A; Figure S3A).

In order to understand whether there is a causal relationship between MSK1 activation and H3S10ph, we electroporated a siRNA/GFP bicistronic construct for MSK1 in the TA muscle and 1 week later performed our stimulation protocol. We found that inhibition of MSK1 by RNAi was sufficient to prevent almost completely H3S10ph after stimulation (Figure 3C,D). It is worth noting that the non‐transfected fibres in the same muscle showed numerous H3S10ph‐positive myonuclei, indicating that the muscle was properly stimulated. Importantly, results obtained by electroporation were confirmed also in MSK1/2 k.o mice, which did not show an increase in H3S10ph after stimulation, despite the activation of p38 (Figure 3E). Next, we determined whether the induction of an SRF target gene was blunted in MSK1/2 k.o. muscles after exercise. Interestingly, we found that the increase seen in *Fos* and *Egr1* transcripts after eccentric contractions is prevented in MSK1/2 k.o. mice (Figure 3F; Figure S3B), strongly suggesting a blunted increase in SRF activity.

Lastly, we wondered whether this SRF‐dependent fingerprint could be important in exercise‐induced muscle remodelling. To address this issue, we analysed protein synthesis rates after eccentric contractions in both wild‐type and MSK1/2 k.o. mice. Wild‐type mice showed a significant increase in protein synthesis rates after stimulation (Figure 3G,H). Surprisingly, this increase in protein synthesis rates was significantly reduced in MSK1/2 k.o. mice, suggesting an important role for SRF‐dependent activity in exercise‐induced increases in protein synthesis. Interestingly, this difference in protein synthesis rates is not linked to a different activation of the Akt‐mTORC1 pathway, a critical mediator in most models of adult muscle growth^2^, ^16^ (Figure S4A‐C).

### 2.5 Exercise induces MAPK and histone phosphorylation also in human skeletal muscle

**Figure 4:**
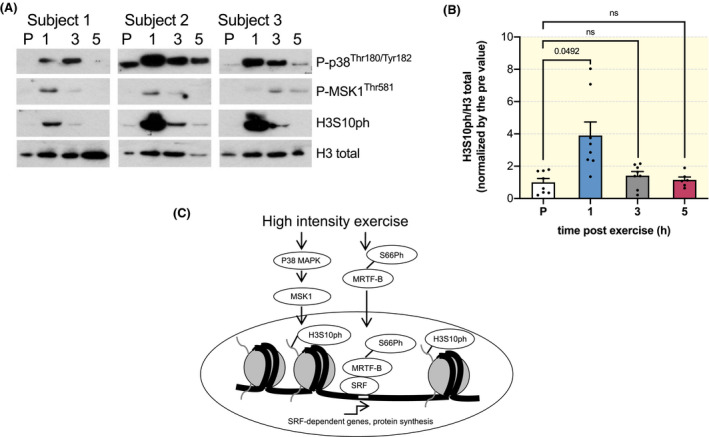
Exercise in humans leads to a rapid increase in MAPK activation and H3S10ph in muscle biopsies. A, Representative blots for three subjects showing the phosphorylation of p38, MSK1, and H3S10 at 1, 3 and 5 h after one bout of high intensity exercise. B, Quantification of the phosphorylation levels H3S10 after one bout of exercise (n = 8, mixed‐effect analysis with Geisser‐Greenhouse correction *P* = .0289; RM one‐way ANOVA, Dunnett's multiple comparison test: PRE vs 1 h *P* = .0492, PRE vs 3 h *P* = .1362 and PRE vs 5 h *P* = .7232. C, scheme of the signalling changes occurring after a single bout of eccentric exercise leading to increased SRF activity through phosphorylation of MRTF‐B and H3S10ph

Lastly, while animal studies are critical to better understand basic signalling mechanisms, eccentric exercise regimes in mice have the important limitation of being forced and not voluntary. Therefore, each signalling model needs to be verified in human muscle biopsies taken before and after high intensity exercise. For this reason we analysed human muscle biopsies, previously characterized by an increase in SRF signalling after high intensity exercise, which lead to an increase in fibre cross sectional area over time.^11^ High intensity exercise leads to a rapid increase in the activation of P38 and MSK1 (Figure 4A). Importantly, this increase in MAPK activation was accompanied by a significant increase in H3S10ph (Figure 4A,B). Unfortunately, an antibody for the detection of human MRTF‐B phosphorylation was not available. Taken together, these results suggest that exercise in human skeletal muscle leads to the activation of the same signalling pathway observed in murine muscle after electrical stimulation.

## 3 DISCUSSION

Exercise is one of the most potent therapeutic approaches in numerous pathologies. Skeletal muscle remodelling, and the subsequent systemic effects, play a major role in mediating the beneficial effects of exercise. Despite this enormous potential of exercise, the mechanisms responsible for exercise‐induced muscle remodelling are still far from clear. Here, we focused on identifying the signalling pathways responsible for stimulating muscle growth after high intensity exercise. Using an unbiased phosphoproteomics screen we found that MRTF‐B is rapidly phosphorylated on Ser66 after a single bout of exercise. This phosphorylation is critical for MTRF‐B nuclear accumulation after exercise and is accompanied by an increase in the transcription of *Fos*, an SRF target gene. Interestingly, the same exercise protocol also leads to the MSK1‐dependent phosphorylation of histone 3, a known transcriptional activator mark, on SRF target genes. Ablation of MSK1 is sufficient to prevent exercise‐dependent increases in histone phosphorylation, induction of SRF target genes, and protein synthesis. Importantly, both the phosphorylation of histone 3 as well as the activation of the MAPK pathway is also observed in muscle biopsies taken from human skeletal muscle after exercise.

### 3.1 High intensity exercise induces activation of the MRTF‐SRF axis

Interestingly, in our phosphoproteomics analysis we found that one of the top‐ranked modifications is linked to alterations in actin dynamics.^4^, ^17^ In most cell types, mechanical cues can rapidly induce changes in the actin cytoskeleton leading to changes in gene transcription.^4^ Specifically, the localization of MRTFs in vitro depends on the level of actin polymerization.^18^ Nuclear‐cytoplasmic shuttling of MRTFs has not been studied in skeletal muscle in the context of exercise in vivo. The importance of these co‐activators for muscle formation is further confirmed by the observation that muscle‐specific deletion of MRTF‐B in MRTF‐A knockout mice leads to the formation of disorganized sarcomeres and perinatal lethality.^6^ Activation of the pathway, by nuclear translocation of MRTFs, has been suggested to be important for muscle remodelling after exercise. Indeed, STARS affects actin polymerization in muscle cells, leading to nuclear translocation of MRTFs, which is required for full activation of SRF‐dependent gene transcription.^19^ Furthermore, STARS expression is strongly increased after exercise^3^ and, importantly, this increase is significantly reduced in ageing subjects, which show a reduced response to resistance‐exercise induced muscle remodelling.^20^ In this manuscript we identified the phosphorylation of Serine 66 on MRTF‐B as being critical for its exercise‐dependent nuclear translocation in vivo. It has been shown that this phosphorylation site on MRTF‐A is important for its nuclear translocation and depends on ERK activation.^21^ Interestingly, ERK phosphorylation is rapidly and significantly increased after exercise,^22^ suggesting it can act as a possible upstream activator of MRTF‐B translocation.

We also found that nuclear accumulation of MRTF‐B is accompanied by an increase in the mRNA levels of SRF‐target genes after eccentric contractions. These findings are in line with our previous work showing that SRF transcriptional activity is significantly increased immediately after various stimuli leading to muscle growth.^2^ Studies performed in mice with an inducible ablation of SRF specifically in adult muscle fibres show that SRF is required for the induction of muscle growth after synergist ablation.^7^ Taken together, our results show that high intensity exercise leads to phosphorylation of MRTF‐B on serine 66, which is required for a complete nuclear translocation, possibly influencing SRF‐dependent gene transcription.

### 3.2 MSK1 is required for H3S10ph and increases in protein synthesis after exercise

Over the past decade many studies showed that a single bout of exercise is able to induce transient increases in mRNA levels of various myogenic, metabolic and regulatory genes, potentially involved in stimulating muscle growth. Although the skeletal muscle transcriptional responses have been extensively studied, our understanding of the epigenetic mechanisms involved in regulating these responses is still rudimentary. Exercise was shown to induce a decrease in DNA methylation and subsequent increases in the transcription of the affected genes.^23^ Other studies looked at histone modifications after exercise, without clearly linking it to the induction of specific genes.^24^, ^25^ Here, we found that eccentric contractions induce activation of p38‐MSK1, which is required for histone 3 phosphorylation, induction of specific SRF target genes, and increases in protein synthesis rates. Interestingly, it was recently shown that H3S10ph is the first step in a cascade of histone modifications leading to activation of SRF target genes.^8^ A similar epigenetic mechanism has been described in the nervous system, where stimuli mimicking neural activity lead to a rapid H3S10ph in hippocampal neurons,^26^ accompanied by an induction of immediate early genes, like *Fos* and *Jun*.^27^ Here, for the first time we showed that H3S10ph occurs on SRF and AP1 target genes in skeletal muscle after a single bout of exercise and this is associated with an increased transcription of SRF target genes. Interestingly, the same stimuli which lead to H3S10ph in hippocampal neurons and subsequent induction of *Fos* and *Jun*, are absent when *Srf* is inducibly deleted from these neurons, linking H3S10ph‐dependent *Fos* transcription directly to SRF activity.^28^ As was observed in neurons and other cell types,^29^ we found that MSK1 is the kinase responsible for H3S10ph in skeletal muscle fibres after eccentric contractions. Interestingly, activity‐dependent synaptic plasticity requires MSK1, possibly through the transcription of the immediate early gene *Arc*, a critical mediator of synaptic modulation.^30^ Also, in skeletal muscle, we observed a significant reduction in protein synthesis rates in MSK1/2 k.o. mice after exercise. Part of the reduced protein synthesis rates could be ascribed to the lack of the induction of immediate early genes, like *Fos* and *Jun*. Indeed, it was shown that JUNB, another member of this transcription factor family is able to rapidly increase muscle size both by decreasing protein breakdown and increasing protein synthesis.^31^

## 4 CONCLUSIONS

Taken together, we found that a single bout of high intensity exercise leads to a rapid activation of MRTF‐SRF signalling, which is accompanied by an MSK1‐dependent chromatin remodelling on SRF target genes. Ablation of MSK1 prevents transcription of SRF target genes, and the subsequent increase in protein synthesis in muscle fibres after exercise. Importantly, the pathway identified in mouse skeletal muscle is also activated in humans immediately after muscle activation, identifying a new signalling fingerprint which is linked to the increase in protein synthesis after exercise.

## 5 MATERIALS AND METHODS

### 5.1 Mice and treatments

Male C57Bl6 mice between 3 and 6 months of age were used, if not stated otherwise. MSK1/2 knockout mice were viable and fertile, without any apparent growth problem, as described previously.^32^ To perform electrical stimulation mice were anaesthetized by an intraperitoneal injection of xylazine (Xilor) (20 mg/Kg) and Zoletil (10 mg/Kg). Next, a small incision was made from the knee to the hip, exposing the sciatic nerve. Before branching of the sciatic nerve, Teflon‐coated 7 multistranded steel wires (AS 632; Cooner Sales, Chatsworth, CA, USA) were implanted with sutures on either side of the sciatic nerve. To avoid recruitment of the ankle dorsal flexors, the common peroneal nerve was cut. Torque production of the plantar flexors after nerve stimulation was measured using a muscle lever system (Model 305C; Aurora Scientific, Aurora, ON, Canada). Protein synthesis was measured using the SUnSET technique^33^ after three rounds of stimulation every other day, leaving mice with ad libitum access to food and water for the whole duration of the experiment. Mice were anaesthetized 24 hours after the last bout of exercise and then, given an intraperitoneal injection of 0.040 μmol/g puromycin dissolved in 100 μL of PBS. At exactly 30 minutes after injection, muscles were collected and frozen in liquid N2 for WB. Controlateral muscles were used as controls to determine puromycin incorporation 24 hours after the last stimulation. In vivo skeletal muscle electroporation was performed in 12‐week‐old wild‐type mice (C57BL/6). TA muscles were transfected as described previously.^34^ Muscles were analysed 14 days after electroporation. Experimental protocols were reviewed and approved by the local Animal Care Committee, University of Padova.

### 5.2 Exercise protocol

For acute exercise, a 600 ms stimulus a 100 Hz was transmitted every 30 seconds for a total of 50 repetitions. The complete protocol lasts about 25 minutes and no sag of force has been reported. Muscle lengthening during contraction was achieved by dorsal flexing of the foot at a velocity of 40 mm/s. The footplate was moved 200 ms after initiation of stimulation train, thus eccentric pull occurs during the isometric plateau of the tetanus. The range of movement during the pull was calculated to be 30°, clearly inside physiological limits of movement for the foot. Force was measured as described previously using a setup from Aurora Scientific.^35^, ^36^ For the protein synthesis stimulation, the stimulation protocol has been repeated three times on day 1, 3 and 5 with muscle taken at day 6. For the treadmill exercise, C57Bl6 mice were subjected to an eccentric running training protocol consisting of running to exhaustion, with a 10 degree decline, at increasing velocity, according to the protocol of exercise previously described.^37^ Briefly, exercise consists in 17 cm/seconds for 40 minutes, 18 cm/seconds for 10 min, 20 cm/seconds for 10 min, 22 cm/seconds for 10 min, and then, increasing velocity of 1 cm/seconds and or 2 cm/seconds alternatively every 5 minutes, until exhaustion. Exhaustion was defined as the point at which mice spent more than 5 seconds on the electric shocker without attempting to resume running.

### 5.3 Immunoblotting and immunohistochemistry

Western blotting was performed as described previously.^16^ The following antibodies were used: Phospho‐H3^Ser10^ (#3377S), Acetyl‐Histone H3^Lys9/Lys14^ (#9677S), H3 (#9715S), P‐MKK3^Ser189^/MKK6^Ser207^ (#9231S), Phospho‐p38 MAPK^Thr180/Tyr182^ (#9211S), P‐MSK1^Thr581^ (#9595S), MSK1 (#3489S), p38 MAPK (#9212S), Phospho‐Akt^Ser473^ (#4060S), Phospho‐S6^Ser240/244^ Ribosomal Protein (#5364S) were from Cell Signalling, c‐MYC (11667149001) was from Roche, GFP (A11122) was from Life technologies, GADPH (Ab8245) and Tri‐methyl‐Histone H3^Lys4^ (Ab71998) were form Abcam, anti‐Puromycin (MABE343) and Phospho(Ser10)‐acetyl (Lys14)‐Histone H3 (07‐081) from Millipore. Actin is from Santa Cruz (sc‐56459). Nuclei were identified with a DAPI staining (Sigma D9542). The secondary antibodies used are CST anti‐Rabbit HRP (#7074S) and Biorad anti‐mouse (#1706516). The bands were visualized by an enhanced chemiluminescence method using the Luminata Classico HRP substrate (WBLUCO500 Millipore). For immunohistochemistry, 10µm tissue sections were treated with PFA 4% (w/v) for 10 minutes at RT, then PBS‐Triton‐X 100 0.1% (w/v), in case with incubation with mouse primary ab, sections were treated with MOM (Vector Labs MKB‐2213) for 40 minutes at RT. We used antibodies specific for P‐H3^S10^ (Rabbit mAb, Cell Signalling #3377S; 1:1000), Dystrophin (MANDRA1‐7A10; DSHB, 1:300), anti‐GFP (Molecular Probes; 1:200) incubated in 0.5% BSA (Sigma A3912) 2% goat serum (Sigma G9023) at 4°C overnight. Secondary antibodies are rabbit 594 (Biorad 111‐585‐144) and mouse 488 (Biorad 115‐485‐166) incubate 1:200 at 37°C for 1 hours in 0.5% BSA 2% goat serum. Image analysis was done in a blinded manner.

### 5.4 Electroporation and plasmids

Experiments were performed in 12‐week‐old wild‐type mice (C57BL/6). TA muscles were transfected as described previously.^34^ Muscles were analysed 14 days after electroporation. Tibialis Anterior muscle were transfected with different plasmids: MRTF‐B‐GFP, MRTF‐B‐(S66A)‐GFP, Histone 2B‐RFP, pZacf‐U6‐luc‐ZsGreen‐MSK1, shLuc pRRL.

### 5.5 Quantitative phospho‐proteomics

Extensive details of methodology can be found in the supplementary methods. Briefly, equal amounts of gastrocnemius muscle tissue protein from the ^13^Lys6 heavy‐labelled stable isotope labelling by amino acids (SILAC) in cell culture mouse,^13^ and a nonlabelled wild type, with or without eccentric stimulation, were mixed (n = 4, both for control and stimulated muscles, muscles taken out immediately at the end of stimulation).

Proteins were digested with LysC and afterwards separated with strong cation exchange chromatography with a subsequent TiO_2_ enrichment. The ratio of labelled to unlabelled peptides was determined by liquid chromatography and tandem MS (LTQ Orbitrap Velos mass spectrometer, Thermo Scientific) and used to identify the ratio of altered phosphopeptides.^38^, ^39^

With the help of the MaxQuant software 1.5.3.8^40^ and its implemented Andromeda search engine the raw files were analysed. Default settings were used with enabling of the match‐between runs and requantify option. Statistical analysis, GO annotations, paired *t*‐tests and data visualization were performed using the Perseus software^41^ and the Instant Clue software.^42^ The mass spectrometry proteomics data have been deposited to the ProteomeXchange Consortium via the PRIDE^43^ partner repository with the dataset identifier PXD016163.

### 5.6 Real time PCR

Total RNA was extracted from gastrocnemius muscles using TRIzol (Invitrogen). Complementary DNA was generated from 0.4 μg of RNA reverse‐transcribed with SuperScript IV Reverse Transcriptase (Invitrogen). cDNA samples were then amplified on the 7900HT Fast Real‐Time PCR System (Applied Biosystems) using the Power SYBR Green RT‐PCR kit (Applied Biosystems). HPRT and GAPDH have been used as housekeeping genes to normalize data. The primers sequence has been reported in Table S1.

### 5.7 In vivo ChIP‐seq

ChIP‐seq was performed with sonicated nuclear extract prepared from formaldehyde‐crosslinked gastrocnemius muscles using anti‐H3S10ph (Active Motif) as described in.^44^ Construction of Illumina ChIP libraries was performed as previously described.^45^ Libraries were run on a HighSeq4000 sequencer (Illumina) at the NGS Core Facility of Helmholtz Zentrum Munich.

For the bioinformatics analyses, FASTQ files were mapped against the mouse mm10 genome and processed as previously described.^45^ Multi mapping reads were removed with bamtools version 2.4.0.^46^ Sequencing read depth was adjusted by down sampling replicate BAM files to the replicate with lowest read count. Macs2 version 2.1.1^47^ peak caller was used to perform peak calling on the replicates using input DNA. Peak calling cutoff was set to *P*‐value .001 using the “broad” setting. Read density distribution (Bedgraphs) were used to visualize ChIP‐seq tracks with Integrated Genome Browser.^48^ A peak table was created for each sample. Replicate sample tables were then combined into a unified peak universe with unique ranges across the genome and containing overlapping peaks information including the tag counts (enrichment score). “Reproducible” peaks were common between both replicate samples. Bedgraphs were used to plot the read density map near the universe peak centres using deepTools version 2.2.4.^49^ Replicates for each factor were merged using UCSC tools bedGraphToBigWig and bigWigMerge (http://hgdownload.cse.ucsc.edu/admin/exe/macOSX.x86_64/).

Then deepTools computeMatrix tool was used followed by plotHeatmap tool.^49^

Peak annotation was performed with HOMER version v4.8.^50^ GENCODE database for mm10^50^ was used as a reference for assigning feature level annotation. Genomic Regions Enrichment of Annotations Tool (GREAT)^51^ was used to analyse functional significance of H3S10ph peaks using mouse NCBI build 38 genome assembly (mm10), whole genome as background, and associating genomic regions with genes using “two nearest genes” and default settings. H3S10ph ChIP‐seq data are available at Gene Expression Omnibus, accession number GSE139661.

### 5.8 Human biopsies

Human skeletal muscle tissue originated from a previous study on exercise‐induced STARS‐SRF signalling, as described in detail previously.^11^ In brief, muscle biopsies for the current study comprise biopsies from eight healthy untrained young men of the original study collected before and at 1, 3 and 5 hours after single‐bout isolated high intensity concentric resistance exercise. Prior to the single‐bout trial, subjects were subjected to a 10‐day period of exercise habituation followed by a 3‐day recovery period. Concentric exercise was conducted in an isokinetic dynamometer (Humac Norm, CSMi Medical Solutions, Stoughton, MA, USA), as six sets of 10 maximal isolated contraction at 30° x sec^−1^ angular velocity, with 1 minute of recovery between sets. For the current subjects A protein + carbohydrate supplement was provided immediately after cessation of the single‐bout exercise protocol. Muscle biopsies were harvested from the middle section of the m. vastus lateralis muscle by use of the Bergström needle technique, with at least 3 cm between incision sites of each biopsy and carefully attempting to reach the identical sampling depth between biopsies. The muscle samples were quickly dissected free of visible fat and connective tissue and stored at −80°C until further investigation.

### 5.9 Statistics

Statistical analysis has been performed using GraphPad Prism software. Sample size is reported in figure legend for each experiment. Animals has been randomly assigned into groups. In all single point data figures values are expressed as means ± SEMs while in all other figures data are means ± SDs values. Differences between groups were assessed using pair or unpaired *t*‐test depending on the nature of the data, ordinary one‐way or two‐way ANOVA, repeated measures (RM) one‐way or two‐way ANOVA with Tukey's, Sidak's or Dunnet's correction for multiple comparisons as suggested by the software and stated in figure legends. Significance was defined as a *P*‐value of <.05. To identify outliers, the data was analysed using two different outliers detection methods: (a) median and median absolute deviation method (MAD) and (b) median and interquartile deviation method (IQD). According to both methods, there was one outlier in the puromycin data‐set, as it fell outside the identified ranges. This value was an outlier in the stimulated MSK1/2 knockout mice. By performing the outliers identification in Prism, the Grubbs test and the ROUT test (Q = 2%) confirmed the same value as the only outlier in the dataset. Furthermore, since we compared control and stimulated muscles from the same animal, we also removed the value from the control muscle taken from the same animal.

## CONFLICT OF INTEREST

Francesca Solagna, Leonardo Nogara, Kenneth A. Dyar, Franziska Greulich, Ashfaq Ali Mir, Alessia Geremia, Martina Baraldo, Clara Turk, Theresa Bock, Roberta Sartori, Jean Farup, Henriette Uhlenhaut, Kristian Vissing, Marcus Krüger, and Bert Blaauw declare that they have no conflict of interest.

## Supporting information

Supplementary Material

Table S2
