# Supplementary material for: Exercise‐dependent increases in protein synthesis are accompanied by chromatin modifications and increased MRTF‐SRF signalling
Source: Acta Physiol (Oxf). 2020 May 30;230(1):e13496. doi: 10.1111/apha.13496 (PMC7507144; doi:10.1111/apha.13496)
Supplement: Supplementary file 1 — Supplementary Material [file APHA-230-e13496-s001.pdf]

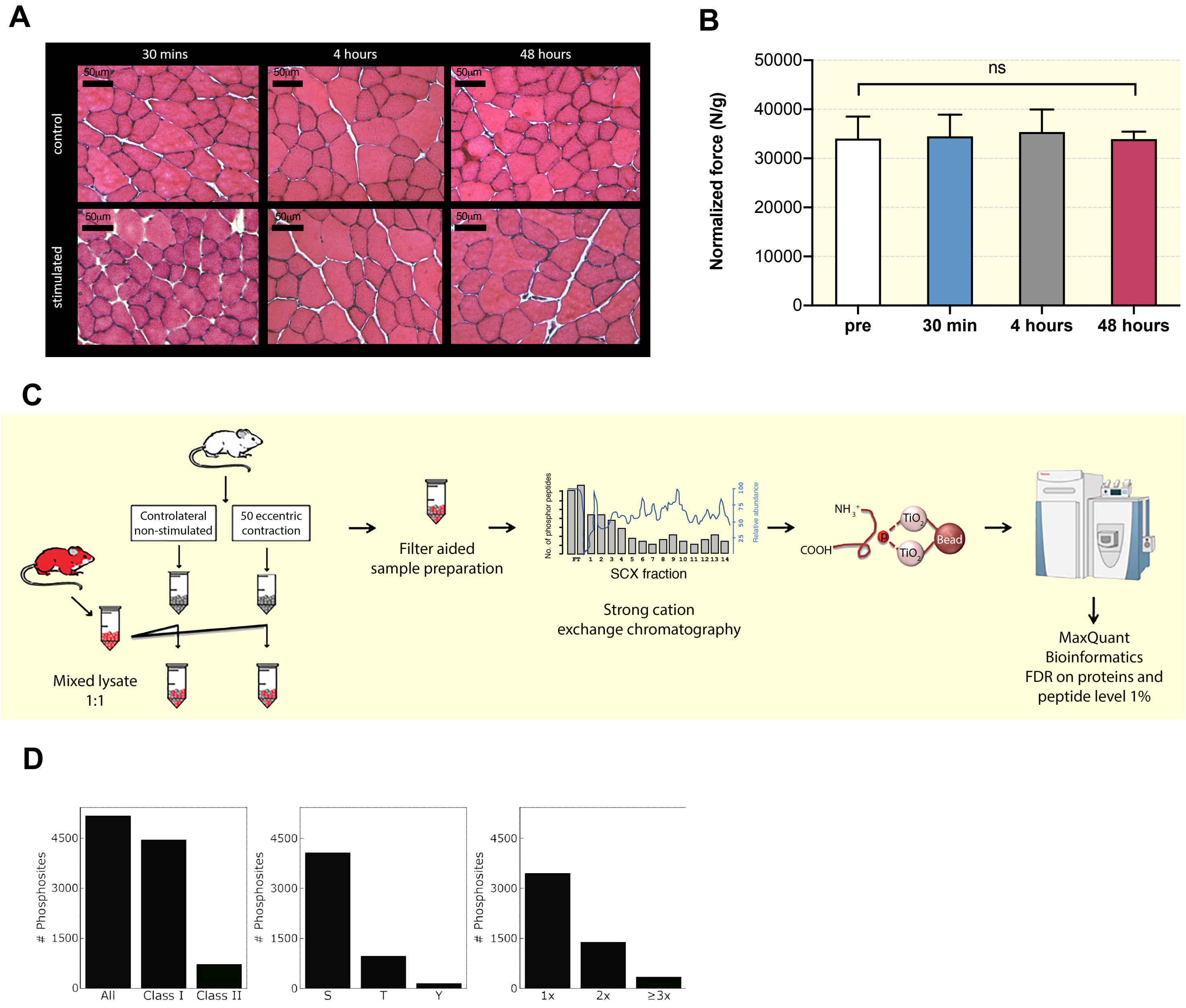

E

Protein names	Gene names	Amino acid	Position	p-value	log2 difference
Transforming acidic coiled-coil-containing protein 2	Tacc2	S	2213	0.04792	3.65
Transforming acidic coiled-coil-containing protein 2	Tacc2	T	2224	0.03264	3.56
Myocardin-related transcription factor B (Mrtfb)	Mrtf-b	S	66	0.06635	3.33
Heat shock 70 kDa protein 4	Hspa4	T	540	0.00546	3.24
Cardiomyopathy-associated protein 5	Cmya5	S	1491	0.03471	3.12
Adenylosuccinate synthetase isozyme 1	Adssl1	S	7	0.00993	2.96
Calpain-3	Capn3	S	611	0.02821	2.92
Calcium/calmodulin-dependent protein kinase type II subunit beta	Camk2b	S	343	0.00054	2.84
Nascent polypeptide-associated complex subunit alpha, muscle-spe	Naca	S	1936	0.03710	2.63
Nascent polypeptide-associated complex subunit alpha, muscle-spe	Naca	S	790	0.04364	2.60
Titin	Ttn	S	1805	0.00001	2.56
CAP-Gly domain-containing linker protein 1	Clip1	S	458	0.01716	2.42
Eukaryotic translation initiation factor 4B	Eif4b	S	497	0.01678	2.42
SH3 and cysteine-rich domain-containing protein 3	Stac3	S	9	0.00373	2.36
CapZ-interacting protein	Rcsd1	S	68	0.00016	2.36
Myosin regulatory light chain 2, skeletal muscle isoform	Mylpf	S	15	0.00043	2.34
Myosin regulatory light chain 2, skeletal muscle isoform	Mylpf	S	16	0.00047	2.34
Myosin regulatory light chain 2, skeletal muscle isoform	Mylpf	T	25	0.02276	2.27
Eukaryotic translation initiation factor 4B	Eif4b	S	504	0.00056	2.26

F

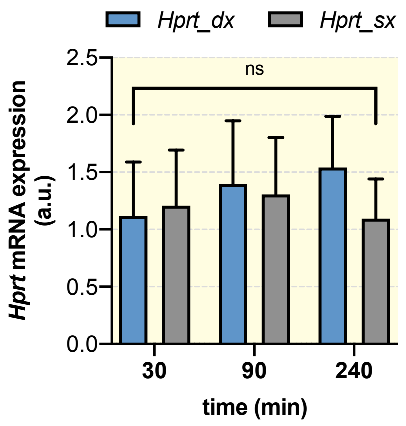

Figure S1. A) H&E staining of muscle sections at different timepoints after stimulation (30 minutes, 4 and 48 hours), with the respective non-stimulated contralateral muscle above. No signs of muscle damage at any timepoint after stimulation. **B)** Maximal tetanic force achieved upon 100Hz electrical stimulation at different time points (30 minutes, 4 and 48 hours). No alteration of force production at different timepoints after the stimulation protocol are reported (n=4 per group, ordinary one-way ANOVA, not significant). **C)** Phosphoproteomics workflow diagram. **D)** Number of quantified phosphorylation-sites grouped in class I (localization probability > 0.75) and class II (localization probability >0.5 and <0.75) sites. Distribution of phosphorylated S, T and Y in the phosphoproteome. Quality of the singly and multiply phosphorylated peptides. **E)** List of top-ranked changed phosphorylation sites after 50 eccentric contractions (p-value<0.07). **F)** *Hprt* mRNA expression level comparison between stimulated (dx) and control (sx) legs used as normalized for Figure 1 panel F (RM two-way ANOVA, matched values, Sidak's multiple comparison test, not significant).

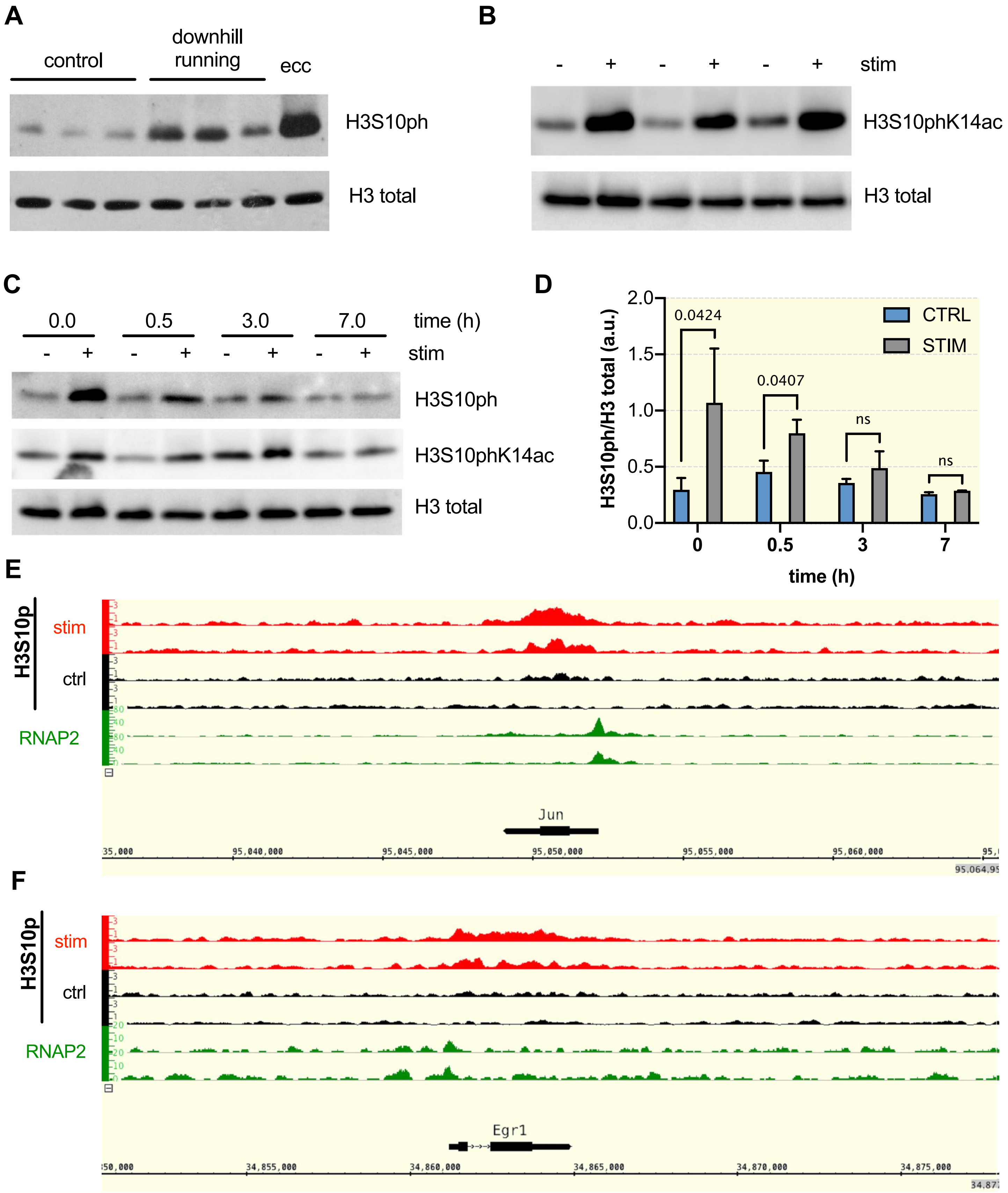

Figure S2. A) Western blot showing the increase in H3S10ph due to downhill running compared with that of eccentric muscle contractions. **B)** Western blot showing the double modification H3S10phK14ac on histone 3 in muscle after eccentric contraction. **C)** Time course of the phosphorylation of H3S10 at different time points (0, 0.5, 3 and 7 hours) after the stimulation protocol. Control is the unstimulated contralateral leg within the same animal. **D)** Quantification of H3S10ph shown in the blot panel S2C (RM two-way ANOVA, interaction P=0.0083, paired t-test). **E)** Genome browser view of *Jun* gene showing H3S10ph enrichment across the gene body. RNA polymerase II (RNAP2) marks the promoter. **F)** Genome browser view of *Egr1* gene showing H3S10ph enrichment across the gene body. RNA polymerase II (RNAP2) marks the promoter.

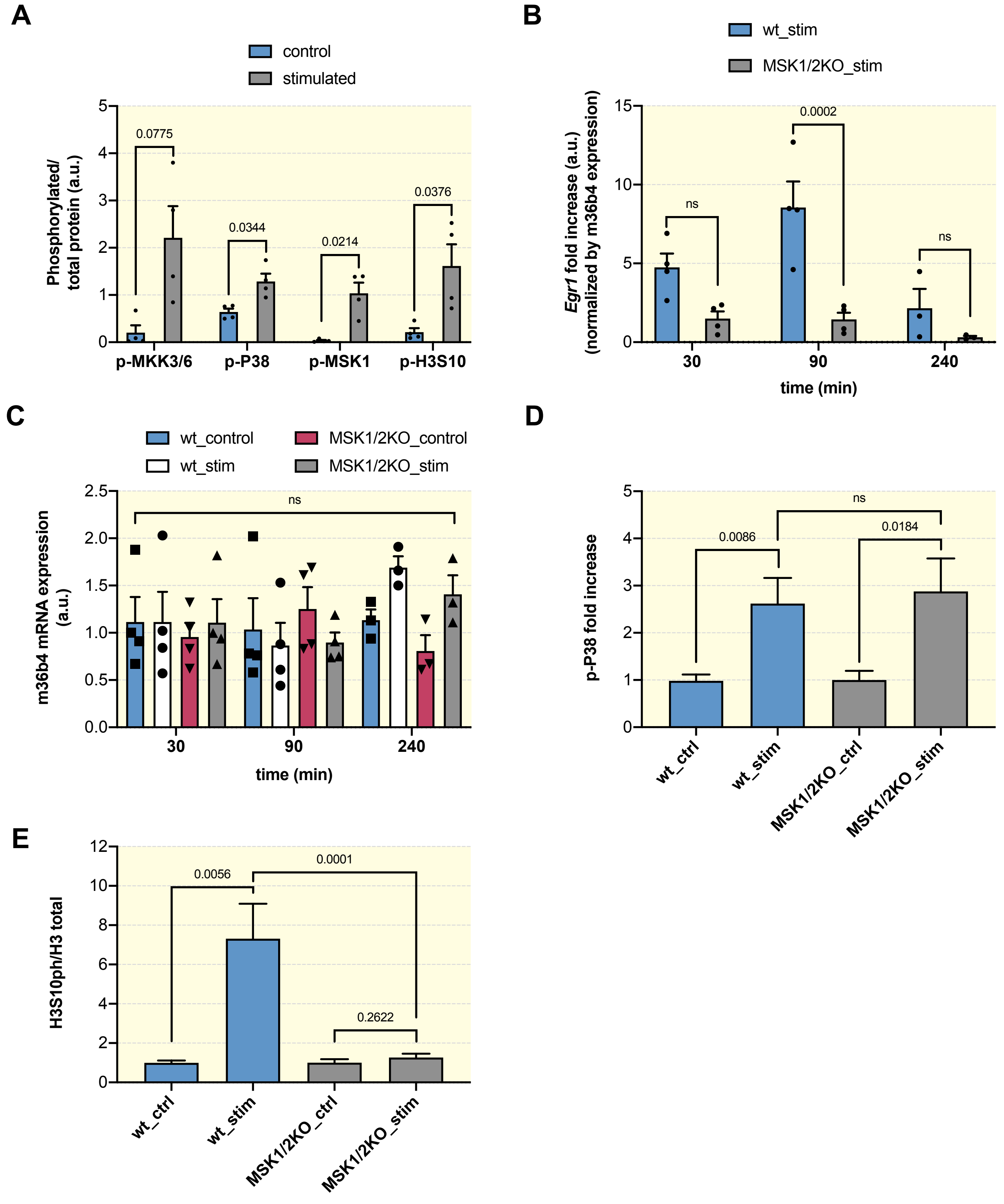

Figure S3. A) Quantifications for the western blot in Figure 3A at one hour after exercise (paired t-test). **B)** Real time results for the transcription of *Egr1* at different time points (0, 90 and 240 minutes) after the eccentric contraction protocol in wt and MSK1/2 ko mice (n=4 wt animals, n=4 MSK1/2ko animals. Mixed effect analysis, interaction P=0.0422, two-way ANOVA, Sidak's multiple comparison test, 30 min comparison P=0.0741, 90min comparison P=0.0002, 240min comparison P=0.5671). **C)** m36b4 mRNA expression level comparison between stimulated (dx) and control (sx) in wt and MSK1/2 ko mice legs used as normalized for Figure 3 panel F (RM two-way ANOVA, not significant). **D)** Quantification of p-P38 in wt and MSK1/2 ko mice stimulated and control legs. (n=17 wt animals, n=14 MSK1/2ko animals. RM Two-way ANOVA, interaction P= 0.7655, stimulation effect P=0.0004, genotype effect P=0.7880, Sidak's multiple comparison test, paired t-test). **E)** Quantification of p-H3S10 in wt and MSK1/2 ko mice stimulated and control legs. (n=21 wt animals, n=20 MSK1/2ko animals. RM Two-way ANOVA, interaction P=0.0051, Sidak's multiple comparison test, ko_ctrl vs ko_stim P=0.9997, paired t-test).

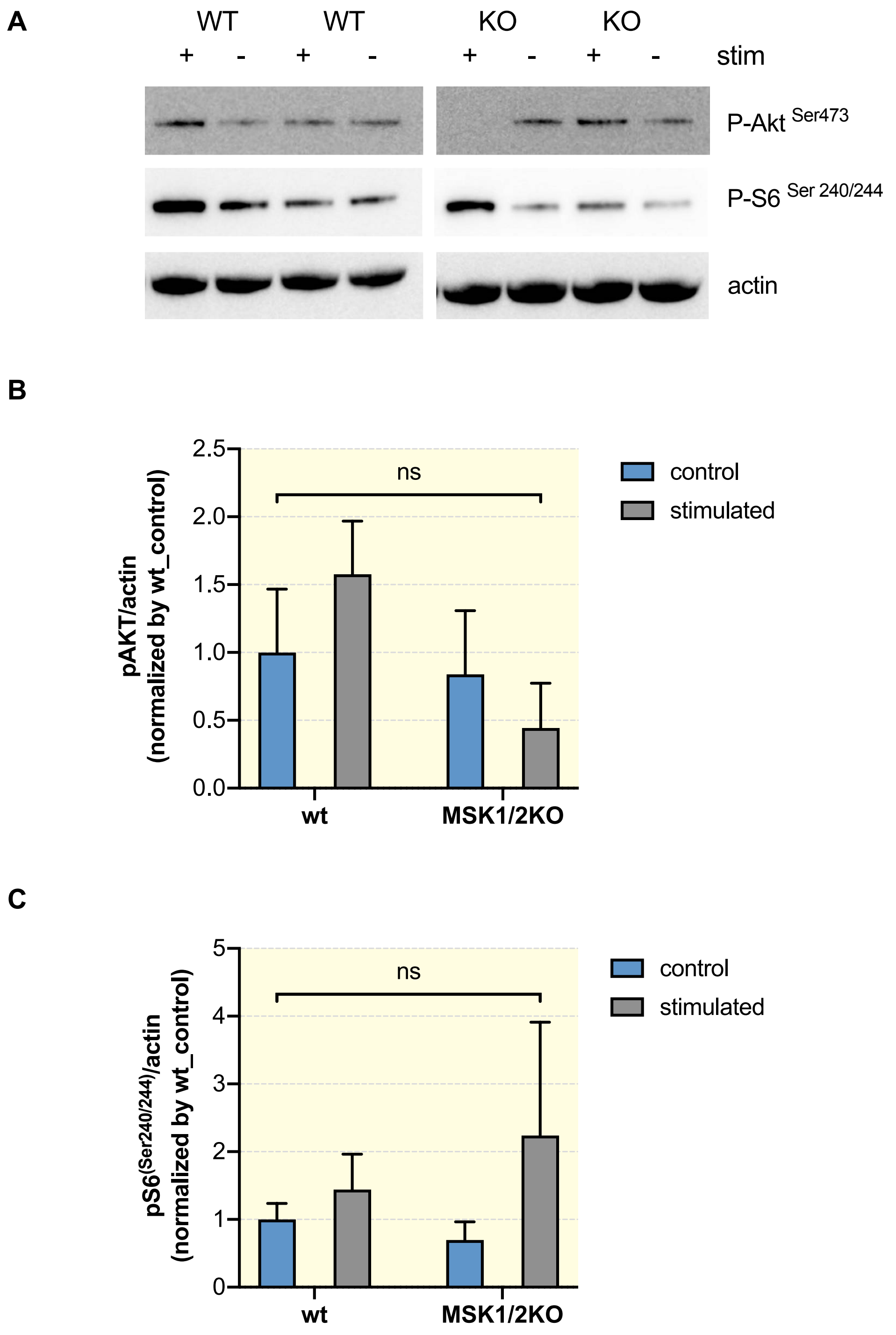

Figure S4. A) Western blot analysis of Phospho-Akt^{Ser473} and Phospho-S6 Ribosomal Protein showed no significant changes in wt and MSK1/2KO animals after three bouts of exercise (day 1, 3 and 5, muscle taken at day 6). **B)** Quantification of Phospho-AktSer^{473} in western blot in panel A (RM two-way ANOVA, interaction P= 0.1662, genotype P=0.2469, stimulation P=0.7797). **C)** Quantification of Phospho-S6 Ribosomal Protein in western blot in panel A (RM two-way ANOVA, interaction P=0.4729).

Table 1 - qPRC primers sequence

FOS for	ACTGAGAAGACTGGATAGAGCC
FOS rev	GCGTTGAAACCCGAGAAC
EGR1 for	AGCGGCGGTAATAGCAGC
EGR1 rev	GATGTCAGAAAAGGACTCTGTGGTC
HPRT for	GTTTGTTGTTGGATATGCCCTTG
HPRT rev	GGCAACATCAACAGGACTCC
m36b4 for	TATGGGATTCGGTCTCTTCG
m36b4 rev	AGCGGTTTTGCTTTTTCATC

Table 1 - qPRC primers sequence

Supplementary Methods

Strong Cation Exchange Chromatography (SCX) and Phosphopeptide enrichment using TiO_2 Beads

Skeletal muscle from non-labeled controls (~3 mg protein lysate) and non-labeled stimulated animals were mixed with the equal amount of age-matched skeletal muscle (~3mg protein lysate) from Lys6 ($^{13}C_6$-Lysine)-labelled SILAC mice as a spike-in standard. To enrich for phosphorylated peptides, ~3 mg of labelled and non-labelled muscle tissue lysates were digested with LysC and fractionated using strong cation exchange chromatography (SCX). Each fraction was subjected to sequential enrichment using titanium dioxide beads (TiO_2) beads to enrich phosphorylated peptides. For protein digestion the FASP method was performed as described previously (Wisniewski et al., 2009). SILAC-mixed protein samples from skeletal muscle lysed in SDS lysis buffer were reduced with 10 mM DTT and heated at 70 °C for 10 min. Next, the samples were loaded onto Amicon Ultra-15 Centrifugal Filter Units (Millipore) equilibrated with 8M urea buffer. SDS in the samples was exchanged with urea buffer, followed by alkylation with 55 mM IAA in the dark. Urea was washed out four times with 50 mM Tris/HCl (pH 8.5). Proteins in the filter were digested overnight with LysC at 30 °C at an enzyme:protein ratio of 1:100. Digested peptides were collected by centrifugation at 4000 *g* for 10 min. The filter was washed once with water to collect remaining peptides.

Peptide solutions were adjusted to a pH of 2.6 with TFA and then 30% ACN, and then loaded onto a 1 mL Resource S column (GE Healthcare) pre-equilibrated with at least five times the column volume of 30% ACN containing 7 mM KH_2PO_4 (pH 2.65). Peptides bound to the column were eluted using a 30-min increasing salt gradient in buffer containing 30% ACN, 7 mM KH_2PO_4 and 350 mM KCl (pH 2.65). The column was washed between runs with a buffer containing 50 mM $K_2HPO_4.3H_2O$ and 500 mM NaCl (pH 7.5). The eluted fractions were pooled based on the chromatogram to obtain a total of nine fractions.

The flow-through and fractions obtained from SCX were vacuum concentrated on a SpeedVac (Eppendorf) and adjusted to 80% ACN/6% TFA (binding buffer condition). The TiO_2 beads were washed with 60% NH_3/40% ACN (elution buffer), followed by 70% ACN to wash out the ammonia, and then with 80% ACN/3% TFA (wash buffer). Washed beads were resuspended in 80% ACN/6% TFA (binding buffer). The flow-through was extracted three times with 5 mg TiO_2 beads and the fractions were extracted twice with 3 mg TiO_2 beads. The multiple extractions of each sample were pooled and loaded onto one layer stacked-C8 StageTips for washing. Beads were washed thrice with binding buffer and thrice with wash buffer. Phosphopeptides were eluted three times from the beads using 25 µL elution buffer. Eluates were vacuum concentrated in a SpeedVac. Concentrated phosphopeptides were acidified using 0.1% formic acid and subjected to LC-MS/MS analysis.

Liquid chromatography and mass spectrometry

Instrumentation for LC-MS/MS analysis consisted of a NanoLC 1000 coupled via a nano-electroionization source to the LTQ Orbitrap Velos mass spectrometer (Thermo Scientific). Peptide separation was carried out on an in-house packed 50 cm (20 cm) column with 1.7 µm (3.0 µm) C18 beads (Dr Maisch GmbH) using a binary buffer system consisting of solution A: 0.1% formic acid (0.5% acetic acid) and B: 80% acetonitrile, 0.1% formic acid (80% acetonitrile, 0.5% acetic acid). Linear gradients from 7–38% B in 150 min were applied with a following increase to 80% B within 5 min and a re-equilibration to 5% B. The resolution for MS spectra was set to 30,000 at 400 mz^{-1} after accumulation of 1E6 ions (AGC target) within a maximal injection time of 60 ms. Top 15 method was applied for CID MS/MS spectra at a resolution of 7,500 at 200 mz^{-1}. AGC target and maximal injection time were set to 1E4 and 30 ms, respectively.
